# Increased Expression of Tissue Factor and Receptor for Advanced Glycation End Products in Peripheral Blood Mononuclear Cells of Patients With Type 2 Diabetes Mellitus with Vascular Complications

**DOI:** 10.1080/15438600490424325

**Published:** 2004

**Authors:** A. E. Buchs, A. Kornberg, M. Zahavi, D. Aharoni, C. Zarfati, M. J. Rapoport

**Affiliations:** 1 Diabetes Unit Internal Medicine “C,” Assaf Harofeh Medical Center Zerifin 70300 Israel; 2 Institute of Hematology Assaf Harofeh Medical Center Zerifin Israel

## Abstract

The aim of the study was to determine the correlation between the expression of tissue factor (TF) and the receptor for advanced glycation end products (RAGEs) and vascular complications in patients with longstanding uncontrolled type 2 diabetes (T2D). TF and RAGE mRNAs as well as TF antigen and activity were investigated in 21 T2D patients with and without vascular complications. mRNA expression was assessed by reverse transcriptase–polymerase chain reaction (RT-PCR) in nonstimulated and advanced glycation end product (AGE) albumin–stimulated peripheral blood mononuclear cells (PBMCs). TF antigen expression was determined by enzyme-linked immunosorbent assay (ELISA) and TF activity by a modified prothrombin time assay. Basal RAGE mRNA expression was 0.2 ± 0.06 in patients with complications and 0.05 ± 0.06 patients without complications (*P* = .004). Stimulation did not cause any further increase in either group. TF mRNA was 0.58 ± 0.29 in patients with complications and 0.21 ± 0.18 in patients without complications (*P* = .003). Stimulation resulted in a nonsignificant increase in both groups. Basal TF activity (U/10^6^ PBMCs) was 18.4 ± 13.2 in patients with complications and 6.96 ± 5.2 in patients without complications (*P* = .003). It increased 3-fold in both groups after stimulation (*P* = .001). TF antigen (pg/10^6^ PBMCs) was 33.7 ± 28.6 in patients with complications, 10.4 ± 7.8 in patients without complications (*P* = .02). Stimulation tripled TF antigen in both groups of patients (*P* = .001). The RAGE/TF axis is up-regulated inT2Dpatients with vascular complications as compared to patients without complications. This suggests a role for this axis in the pathogenesis of vascular complications in T2D.


*Experimental Diab. Res.*, 5:163–169, 2004
ISSN: 1543-8600 print / 1543-8619 online
DOI: 10.1080/15438600490424325

Taylor & Francis
Taylor & Francis Group

### Increased Expression of Tissue Factor and Receptor for Advanced Glycation End Products in Peripheral Blood Mononuclear Cells of Patients With Type 2 Diabetes Mellitus with Vascular Complications

**A. E. Buchs,[1] A. Kornberg,[2] M. Zahavi,[2] D. Aharoni,[1] C. Zarfati,[1] and M. J. Rapoport[1]**

[1]*Diabetes Unit, Internal Medicine "C," and* [2]*Institute of Hematology, Assaf Harofeh Medical Center, Zerifin, Israel*

**The aim of the study was to determine the correlation between the expression of tissue factor (TF) and the receptor for advanced glycation end products (RAGEs) and vascular complications in patients with longstanding uncontrolled type 2 diabetes (T2D). TF and RAGE mRNAs as well as TF antigen and activity were investigated in 21 T2D patients with and without vascular complications. mRNA expression was assessed by reverse transcriptase–polymerase chain reaction (RT-PCR) in nonstimulated and advanced glycation end product (AGE) albumin–stimulated peripheral blood mononuclear cells (PBMCs). TF antigen expression was determined by enzyme-linked immunosorbent assay (ELISA) and TF activity by a modified prothrombin time assay. Basal RAGE mRNA expression was 0.2 ± 0.06 in patients with complications and 0.05 ± 0.06 patients without complications ($P = .004$). Stimulation did not cause any further increase in either group. TF mRNA was 0.58 ± 0.29 in patients with complications and 0.21 ± 0.18 in patients without complications ($P = .003$). Stimulation resulted in a nonsignificant increase in both groups. Basal TF activity (U/$10^6$ PBMCs) was 18.4 ± 13.2 in patients with complications and 6.96 ± 5.2 in patients without complications ($P = .003$). It increased 3-fold in both groups after stimulation ($P = .001$). TF antigen (pg/$10^6$ PBMCs) was 33.7 ± 28.6 in patients with complications, 10.4 ± 7.8 in patients without complications ($P = .02$). Stimulation tripled TF antigen in both groups of patients ($P = .001$). The RAGE/TF axis is up-regulated in T2D patients with vascular complications as compared to patients without complications. This suggests a role for this axis in the pathogenesis of vascular complications in T2D.**

**Keywords** Complications; RAGE; Tissue Factor; Type 2 Diabetes

There is increasing evidence that in addition to hyperglycemia, other factors such as advanced glycation end products (AGEs) and their receptors may be related to the development of these complications. Kilhovd and colleagues demonstrated that in comparison to controls without diabetes mellitus, AGE is elevated in serum of patients with type 2 diabetes (T2D) suffering from ischemic heart disease [1]. AGE deposits were found in mesangial cells from patients with diabetic nephropathy, and retinal Müller cells from patients with proliferative diabetic retinopathy [2, 3]. More so, receptor-facilitated cellular uptake of AGEs induces proliferation of endothelial cells and migration of smooth muscle cells in atherosclerotic lesions [4]. The receptor for AGEs, RAGE, is the most widely researched member of a family of 5 different receptors [5]. It has been detected in cells involved in diabetic complications, such as monocytes, macrophages, and endothelial cells. It was also found in macro- and microvascular lesions, including the aorta, the coronary arteries, and leg arteries, as well as in the retina and glomeruli [6–10]. Moreover, infusion of a truncated

The authors thank Dalia Usi for her assistance in recruiting patients. This work was supported by a grant of the Israeli Diabetes Association.

Address correspondence to Dr. Andreas E. Buchs, Department of Internal Medicine "C," Assaf Harofeh, Zerifin 70300, Israel. E-mail: abuchs@netvision.net.il form of RAGE into diabetic mice with accelerated atherosclerosis reduced AGE uptake into mesangial cells and prevented neovascularization [11–14]. The exact intracellular molecular mechanism of AGE induced macro- and microvascular damage is still unclear, but could be linked to AGE-induced tissue factor (TF) activation, as demonstrated by Bierhaus and colleagues [15]. Incubation of cultured endothelial cells with AGEs induced transcription of TF mRNA and protein expression mediated by the p38 mitogen-activated protein kinase and consecutively the nuclear transcription factor NF-kappa B, which activated the RAGE gene [15–17]. TF is a procoagulant glycolipoprotein that resides in the cellular membranes of monocytes, macrophages, and endothelial cells [18]. It is the main activator of the coagulation system and has been associated with the development of diabetic vascular complications [19]. Increased plasma TF activity, most probably generated by monocytes and endothelial cells, was found in patients with type 1 diabetes suffering from nephropathy [20]. Foamy monocytes and macrophages accumulate in subendothelial cells at an early stage of the development of vascular atheroma and contain large amounts of TF [21]. The existence of fibrin, platelets, and thrombin in atheromas indicates that TF from monocytes-macrophages activates locally the coagulation system. Moreover, thrombin, generated by TF, has a mitogenic effect and causes migration and proliferation of fibroblasts in atheromas [22]. Thus, it appears that the interaction between the AGE-RAGE system and TF in monocytes plays a significant role in the development of vascular complications in diabetes mellitus.

Based on these data, we studied the capacity of peripheral mononuclear blood cells (PBMCs) from patients with poorly controlled, longstanding T2D, with and without complications, to express TF/RAGE before and after stimulation with AGEs. Our findings indicate that TF mRNA and protein expression, as well as expression of RAGE mRNA, are increased in patients suffering from vascular complications.

### PATIENTS AND METHODS

### Patients

Clinical and demographic characteristics of patients are presented in Table 1. Twenty-one patients (13 males and 8 females, age range 41 to 82 years) with longstanding uncontrolled T2D (>20 years; hemoglobin $A_{1C}$ ($HbA_{1C}$) > 8% on repeated determinations, upper normal limit 6.4%) were recruited for the study. Eleven patients suffered from microvascular complications defined as diabetic retinopathy and nephropathy. Ten of these patients also had at least one macrovascular complication, i.e., chronic ischemic heart disease, proven by coronary angiography or coronary bypass graft surgery. Patients were excluded if they suffered from uncontrolled hypertension (>130/80 mm Hg) or if their cholesterol and triglyceride levels exceeded the recommendations of the American Diabetes Association [23]. An entire medical history was obtained and all patients underwent a complete physical examination. Microvascular complications were excluded by a normal fundoscopy, urinary microalbumin excretion <30 mg/24 h in 2 repeated determinations or urinary microalbumin (mg)/creatinine (mg) ratio <0.02, and

**TABLE 1**
Demographic and clinical characteristics of studied patients

| | Complications | No complications | Significance |
|---|---|---|---|
| Number of patients (m/f) | 11 (6/5) | 10 (7/3) | n.s. |
| Duration DM (years) | 22 ± 8 | 28 ± 9 | n.s. |
| Age | 59 ± 8 | 62 ± 13 | n.s. |
| BMI | 27.8 ± 3.5 | 27.2 ± 3.2 | n.s. |
| CIHD | 9/11 | 0/10 | |
| CVA | 4/11 | 0/10 | |
| PVD | 4/11 | 0/10 | |
| $HbA_{1C}$ (%)* | 9.4 ± 1.8 | 8.7 ± 1.6 | n.s. |
| LDL (mg%) | 103 ± 22 | 112 ± 15 | n.s. |
| TG (mg%) | 135 ± 80 | 108 ± 57 | n.s. |
| HDL (mg%) | 53 ± 12 | 58 ± 13 | n.s. |
| Hypertension | 8/11 | 3/10 | n.s. |
| Smokers | 1/11 | 1/10 | n.s. |
| Statin treatment | 5/11 | 2/10 | n.s. |
| Insulin treatment | 5/11 | 6/10 | n.s. |

*Normal value <6.4%.

the absence of symptomatic peripheral neuropathy. Macrovascular disease was considered to be unlikely in patients without microvascular disease in the presence of a normal physical examination, a normal electrocardiogram, and a negative history of coronary artery disease, stroke, or peripheral vascular disease. The local Helsinki committee approved this study and informed consent was obtained from all patients according to the regulations.

### Isolation and Stimulation of PBMCs with AGE Albumin

Twenty milliliter of peripheral blood was collected from an antecubital vein into vacutainers containing EDTA. PBMCs were separated over a Ficoll-Hypaque gradient (Pharmacia), washed, and resuspended in RPMI (Rhenium) containing 10% fetal calf serum and L-glutamine (Biological Industries, Beth HaEmek, Israel). Based on reports by other investigators who tested the effect of AGEs on endothelial cells, AGE albumin and human serum albumin were purchased from Sigma (substitution 2.7 moles hexose/mole protein) and incubated according to their published "peak effect" of 12 to 24 hours [15]. Based on preliminary dose-response studies with different glycated human serum albumin concentrations (5 $\mu$g/mL AGE albumin did not stimulate TF activity and 200 $\mu$g/mL AGE albumin did not increase TF activity any further than stimulation with 50 $\mu$g/mL), $2 \times 10^6$ PBMCs per vial, equalized per monocyte counts, were incubated for 2 and 18 hours in endotoxin-free medium containing 50 $\mu$g/mL glycated human serum albumin or control medium containing 50 $\mu$g/mL human serum albumin. Exposure of 2 hours did not induce a detectable increase in TF or RAGE mRNA below the assay detection limit. After incubation, PBMCs were washed twice in phosphate-buffered saline (PBS), separated into 2 vials for determination of TF activity and mRNA extraction, and frozen at −70°C.

### Determination of Tissue Factor Activity

Frozen cells were thawed at 37°C, resuspended in 1 mL PBS, pH 7.35, and sonicated with ultrasonic vibrator for 60 seconds in an ice bath. TF activity was measured by a modified prothrombin time assay [24]: Pooled anticoagulated plasma, 0.1 mL, from at least 10 normal donors was incubated with 0.1 mL cell suspension at 37°C for 1 minute, 0.1 mL of 0.025 M $CaCl_2$ was added, and clotting time was recorded. Each sample was run in duplicate. TF activity was expressed in units per $10^6$cells, calculated from standard curves of the logarithm of the activity of serial dilutions of standard TF. Dilutions of 1:1024 TF (170 seconds) and 1:16 (32 seconds) were equal to 1 and 64 U, respectively.

### Determination of Tissue Factor Antigen

TF antigen was measured with the Imubid TF ELISA KIT (American Diagnostica, Greenwich, CT) according to the manufacturer's recommendations and expressed in picogram per $10^6$ PBMCs. TF activity and antigen were also calculated per milligram of protein cell lysate: the results were similar.

### Isolation of RNA

PBMCs were lysed in TRI reagent (Sigma) according to the manufacturer's instructions. RNA was resuspended in 10 $\mu$L of DEPC water. Typically 20 to 50 $\mu$g of total RNA was extracted from 20 mL of peripheral blood.

### mRNA Expression of TF and RAGE

Reverse transcription–polymerase chain reaction (RT-PCR) was performed with commercially available kits (Epicentre and Sigma) according to the manufacturer's instructions. Primers for transcription factor, RAGE, and glyceraldehyde-phosphate dehydrogenase (GAPDH) were designed according to previous reports [15, 25]. The coding strand primer for tissue factor corresponds to positions 759 to 784 of the published DNA sequence, the noncoding strand to positions 1016 to 1041. The coding strand primer for GAPDH corresponds to positions 562 to 582, the noncoding strand to positions 807 to 827. The coding strand primer for RAGE corresponds to positions 29 to 54, the noncoding strand to positions 481 to 509. PCR reactions were performed with TAQ polymerase (MBI, Fermentas): 94°C 3 minutes (hot start), 94°C 1 minute, 60°C 30 seconds, 72°C 1 minute, 40 cycles (optimal amplification rate according to preliminary experiments). RAGE was identified by restriction enzyme digestion with BAMH1, revealing 2 DNA fragments of 251 and 188 base pairs, respectively. Ten-microliter aliquots of the PCR reactions were analyzed on a 1.5% agarose gel containing ethidium bromide. For semiquantitative RT-PCR, the signals obtained for tissue factor and RAGE were compared by densitometry to the intensity of the amplified housekeeping gene GAPDH and expressed as optical density (OD) ratio ± SD.

### Statistics

Clinical and demographic characteristics of patients were evaluated by Pearson chi square and Fisher exact tests (1-tail and 2-tail). Analysis of variance (ANOVA) with repeated measures was used for evaluation of the data. The square root transformation was used for those variables that did not have a Gaussian distribution to analyze the effect of stimulation and interaction between the groups.

**TABLE 2a**
Expression of TF and RAGE mRNA in type 2 diabetes patients with and without complications

| | TF RNA OD ratio | | | RAGE RNA OD ratio | | |
|---|---|---|---|---|---|---|
| | Basal | Stimulation | N | Basal | Stimulation | N[c] |
| Complications | 0.58 ± 0.29 | 0.79 ± 0.21 | 11 | 0.2 ± 0.06 | 0.2 ± 0.09 | 10 |
| No complications | 0.21 ± 0.18 | 0.46 ± 0.32 | 10 | 0.04 ± 0.06 | 0.13 ± 0.13 | 10 |
| *P* value | .003[a] | n.s.[b] | | .01[a] | n.s.[b] | |

*Note*. Nonstimulated and AGE-stimulated PBMCs were analyzed. mRNA expression was determined by RT-PCR, quantified by densitometry, and expressed as the ratio of gene/GAPDH mean ± SD. *P* values: [a]Difference between the basal, nonstimulated values in patients with and without complications. [b]Comparison of the response to stimulation of both groups.[c]Comparison between nonstimulated and stimulated values was available for 8 patients within each group.

### RESULTS

### TF mRNA Expression

Basal and stimulated TF mRNA expression was determined in PBMCs from 11 patients with and 10 patients without macro- and microvascular complications. Overall, mRNA TF expression was higher in the studied patients suffering from diabetes than in controls without diabetes (data not shown). As demonstrated in Table 2a, TF mRNA was significantly higher in the complicated than noncomplicated patients: 0.58 ± 0.29 and 0.21 ± 0.18, respectively ($P = .003$). In order to investigate the effect of glycated albumin on TF mRNA expression, PBMCs were incubated for 18 hours in the presence of 50 $\mu$g/mL AGE albumin. This caused a nonsignificant increase in TF mRNA expression in both groups: 0.79 ± 0.21 and 0.46 ± 0.32, respectively.

These data show that TF mRNA expression is increased in patients with T2D and vascular complications.

### TF Antigen and Activity

To determine the relevance of increased TF mRNA expression in complicated patients, we examined the expression of the TF antigen and its functional activity (Table 2b). Basal nonstimulated TF protein expression was 33.7 ± 28.6 pg/$10^6$ PBMCs and 10.4 ± 7.8 pg/$10^6$ PBMCs in patients with and without complications, respectively, $P = .02$. Incubation with AGE albumin (50 $\mu$g/mL) increased TF antigen approximately 3-fold in both groups with diabetes to 108.4 ± 39 pg/$10^6$ PBMCs and 45.5 ± 33.3 pg/$10^6$ PBMCs in the complicated and the noncomplicated groups, respectively. This stimulatory effect on TF antigen was highly significant ($P < .001$).

As shown in Table 2b, basal TF activity was significantly higher in the patients with complications: 18.4 ± 13 U/$10^6$ PBMCs and 6.96 ± 5.2 U/$10^6$ PBMCs, respectively ($P = .003$). Incubation with 50 $\mu$g/mL AGE albumin resulted in a 3-fold increase in TF activity of 56.4 ± 26.2 U/$10^6$ PBMCs and 19.4 ± 7.6 U/$10^6$ PBMCs in groups with and without complications, respectively. The effect of stimulation was significant in both groups ($P = .001$).

These data demonstrate that, in comparison to diabetic individuals without complications, both TF antigen expression and activity are markedly increased in patients with diabetic complications.

### RAGE mRNA Expression

We examined basal and stimulated RAGE mRNA expression to determine whether the increased TF expression and activity is due to AGE-mediated activation of RAGE. As shown in

**TABLE 2b**
Expression and activity of TF in type 2 diabetes patients with and without complications

| | TF activity U/$10^6$ PBMCs | | | TF protein pg/$10^6$ PBMCs | | |
|---|---|---|---|---|---|---|
| | Basal | Stimulation | N | Basal | Stimulation | N[c] |
| Complications | 18.4 ± 13 | 56.4 ± 26.2 | 10 | 33.7 ± 28.6 | 108.4 ± 39 | 8 |
| No complications | 6.96 ± 5.2 | 19.4 ± 7.62 | 6 | 10.4 ± 7.8 | 45.5 ± 33.3 | 5 |
| *P* value | .003[a] | .001[b] | | .02[a] | .001[b] | |

*Note*. Nonstimulated and AGE-stimulated PBMCs were analyzed. TF activity was determined by a modified prothrombin time assay and expressed in arbitrary units/106 PMBCs ± SD. TF protein was determined by ELISA. *P* values: [a]Difference between the basal, nonstimulated values in patients with and without complications. [b]Comparison of the response to stimulation of both groups.

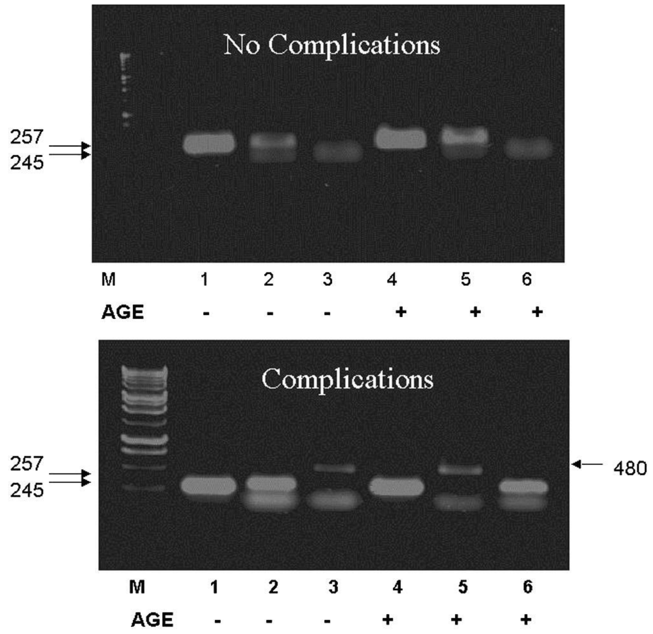

**FIGURE 1**
Representative agarose gels demonstrating TF and RAGE expression nonstimulated and AGE-stimulated PBMCs were analyzed. PCR products were analyzed on ethidium bromide–stained 1.5% agarose gels. DNA marker, left lane (M), fragment sizes (*arrows*): GAPDH: 257 bP; TF: 245 bP; RAGE: 480 bP. Lanes 1 to 3: no stimulation; lanes 4 to 6 stimulation with AGE 50 ($\mu$g/mL). *Top gel*: A typical gel from a patient without complications. GAPDH: lanes 1 and 4; TF: lanes 2 and 5; RAGE: lanes 3 and 6. *Bottom gel*: A typical gel from a patient with complications. GAPDH: lanes 1 and 4; TF: lanes 2 and 6; RAGE: lanes 3 and 5.

Figure 1 and Table 2a, the mean nonstimulated RAGE expression were 0.20 ± 0.06 and 0.04 ± 0.06 in the complicated and the noncomplicated group, respectively, $P < .01$. Stimulation with AGE albumin at 50 $\mu$g/mL failed to cause a significant increase in RAGE mRNA expression in either group.

These data show that mRNA expression is increased in patients with complications in comparison to patients without complications and cannot be further increased by exposure to AGE albumin.

### DISCUSSION

We demonstrate here that nonstimulated and AGE-stimulated TF mRNA and protein expression and TF protein activity are significantly increased in PBMCs from T2D patients with complications as compared to patients without complications. mRNA expression of RAGE is also markedly increased in the former group. To the best of our knowledge, this is the first report correlating existing diabetic complications to the expression of TF and RAGE in PBMCs from patients with diabetes. So far, in clinical settings, TF and RAGE expression has only been examined separately and independently. Ichikawa and colleagues reported increased TF protein expression in monocytes from patients suffering from diabetic nephropathy [26]. Reverter and colleagues noted that serum from patients with type 1 diabetes induced higher TF secretion in normal mononuclear cells than control serum from healthy controls [27]. Serum from patients who developed retinopathy upon long-term follow-up induced TF expression to an especially high level [28]. Jinnouchi and Bierhaus and their colleagues reported RAGE-dependent induction of TF in cultured endothelial cells and accumulation of AGEs in macrophages and foam cells of atheromatous lesions [15, 20]. We therefore investigated whether AGEs also induce TF expression in PBMCs from diabetic patients. Our data further support the role of an overexpressed RAGE/TF cascade in the pathogenesis of vascular complications in diabetes. How the increased TF expression/activity in diabetic patients underlies their complications is not clear. Increased TF, as evident in our patients with complications, may be a facilitating factor for vascular complications by contributing to the hypercoagulable state of diabetes [29]. It is of interest that TF is increased in diabetic individuals without complications in comparison with normal controls, the level of TF of whom we have repeatedly verified to be below the assay detection limit. This raises the possibility of a threshold necessary for the development of vascular complications in T2D patients. RAGE mRNA expression was also higher in patients with complications than in those without. Because activated RAGE is a known mediator of TF expression, this accentuates the importance of increased TF expression/activity in these patients. If this is the case, TF mRNA expression or protein activity could serve as a prognostic marker to define patients who are prone to develop vascular complications. Long-term follow-up studies may allow us to determine whether the association between TF/RAGE expression is coincidental or causal.

In contrast to TF, we could not demonstrate a further AGE-stimulated increase in RAGE mRNA expression in patients with complications. Several mechanisms may explain this discrepancy: (1) We examined the expression of only 1 out of 5 known AGE receptors. The stimulated increase in TF in our patients may be mediated by AGE binding to a different AGE receptor, such as the macrophage scavenger receptor as previously reported in the murine macrophage cell line RAW 264.7 [30, 31]. This hypothesis is supported by He and colleagues, who, despite high serum AGE levels, failed to detect up-regulation of RAGE mRNA or its protein in PBMCs from diabetic patients with macrovascular complications [32]. (2) Increased levels of $HbA_{1C}$ as seen in our patients reflect elevated concentration of AGE in their serum. This may have caused maximal in vivo stimulation of RAGE, preventing further increase by addition of AGEs in vitro. (3) mRNA stability is a key factor determining its levels as shown in endotoxin stimulated mononuclear cells [33]. Thus, it is conceivable that AGEs regulate RAGE mRNA levels in diabetic PBMCs by modifying RAGE stability rather than increasing its synthesis de novo.

To the best of our knowledge, it has not been shown that vascular complications in diabetes are more prevalent or severe in a particular gender. All of our female patients within the study were postmenopausal and not on hormonal replacement therapy. We decided, therefore, not to study male and female patients separately but cannot exclude that this is a topic that will have to be studied in the future. Furthermore, we cannot exclude that some of the "noncomplicated" patients included in our study in fact do have minor asymptomatic macrovascular disease. More sophisticated and invasive tests are required to exclude these conditions. Based on previous reports, however, we considered it unlikely that the included patients suffer from significant cardiovascular disease in the absence of increased microalbuminuria [34]. Hypertension and hyperlipidemia are accepted risk factors for macrovascular disease in diabetes [35]. This is of a particular importance in as much as both hypertension and treatment with statins were more prevalent in the complication group. In addition, more patients with complications took ACE inhibitors or angiotensin II (ATII) antagonists than in the group without complications. However, it is unlikely that these differences explain our results because blood pressure was well controlled in all patients and lipid levels were similar in both groups irrespective of drug treatment. Furthermore, ACE inhibitors and statins reduce rather than increase TF expression in monocytes [36]. We believe, therefore, that these minor differences in risk stratification and drug regimens did not influence our data.

In conclusion, our data demonstrate that diabetic complications are associated with enhanced TF mRNA expression and protein activity as well as increased RAGE mRNA expression. In addition to tight blood glucose control, the RAGE/TF axis could serve as potential treatment target for the prevention of diabetic complications.

### REFERENCES

[1] Kilhovd, B., Berg, T., Birkeland, K., Thorsby, P., and Hanssen, K. (1999) Serum levels of advanced glycation end products are increased in patients with type 2 diabetes and coronary heart disease. *Diabetes Care,* **22,** 1543–1548.

[2] Sugiyama, S., Miyata, T., Horie, K., Iida, Y., Tsuyuki, M., Tanaka, H., and Maeda, K. (1996) Advanced glycation end-products in diabetic nephropathy. *Nephrol. Dial. Transplant*, **11 (Suppl 5),** 91–94.

[3] Hirata, C., Nakano, K., Nakamura, N., Kitagawa, Y., Shigeta, H., Hasegawa, G., Ogata, M., Ikeda, T., Sawa, H., Nakamura, K., Ienaga, K., Obayashi, H., and Kondo, M. (1997) Advanced glycation end products induce expression of vascular endothelial growth factor by retinal Muller cells. *Biochem. Biophys. Res. Commun.,* **236,** 712–715.

[4] Higashi, T., Sano, H., Saishoji, T., Ikeda, K., Jinnouchi, Y., Kanzaki, T., Morisaki, N., Rauvala, H., Shichiri, M., and Horiuchi, S. (1997) The receptor for advanced glycation end products mediates the chemotaxis of rabbit smooth muscle cells. *Diabetes,* **46,** 463–472.

[5] Schmidt, A., Yan, S., Wautier, J., and Stern, D. (1999) Activation of receptor for advanced glycation end products: A mechanism for chronic vascular dysfunction in diabetic vasculopathy and atherosclerosis. *Circ. Res.,* **84,** 489–497.

[6] Schmidt, A., Hori, O., Cao, R., Yan, S., Brett, J., Wautier, J., Ogawa, S., Kuwabara, K., Matsumoto, M., and Stern, D. (1996) RAGE: A novel cellular receptor for advanced glycation end products. *Diabetes,* **45 (Suppl 3),** S77–S80.

[7] Soulis, T., Thallas, V., Youssef, S., Gilbert, R., McWilliam, B., Murray-McIntosh, R., and Cooper, M. (1997) Advanced glycation end products and their receptors co-localise in rat organs susceptible to diabetic microvascular injury. *Diabetologia,* **40,** 619–628.

[8] Wautier, J., and Guillausseau, P. (1998) Diabetes, advanced glycation end products and vascular disease. *Vasc. Med.,* **3,** 131–137.

[9] Sun, M., Yokoyama, M., Ishiwata, T., and Asano, G. (1998) Deposition of advanced glycation end products (AGE) and expression of the receptor for AGE in cardiovascular tissue of the diabetic rat. *Int. J. Exp. Pathol.,* **79,** 207–222.

[10] Ritthaler, U., Deng, Y., Zhang, Y., Greten, J., Abel, M., Sido, B., Allenberg, J., Otto, G., Roth, H., Bierhaus, A, et al. (1995) Expression of receptors for advanced glycation end products in peripheral occlusive vascular disease. *Am. J. Pathol.,* **146,** 688–694.

[11] Park, L., Raman, K., Lee, K., Lu, Y., Ferran, L. J., Chow, W., Stern, D., and Schmidt, A. (1998) Suppression of accelerated diabetic atherosclerosis by the soluble receptor for advanced glycation endproducts. *Nat. Med.,* **4,** 1025–1031.

[12] Wautier, J., Zoukourian, C., Chappey, O., Wautier, M., Guillausseau, P., Cao, R., Hori, O., Stern, D., and Schmidt, A. (1996) Receptor-mediated endothelial cell dysfunction in diabetic vasculopathy. Soluble receptor for advanced glycation end products blocks hyperpermeability in diabetic rats. *J. Clin. Invest.,* **97,** 238–243.

[13] Kislinger, T., Tanji, N., Wendt, T., Qu, W., Lu, Y., Ferran, L., Taguchi, A., Olson, K., Bucciarelli, L., Goova, M., Hofmann, M., Cataldegirmen, G., D'Agati, V., Pischetsrieder, M., Stern, D., and Schmidt, A. (2001) Receptor for advanced glycation end products mediates inflammation and enhanced expression of tissue factor in vasculature of diabetic apolipoprotein E-null mice [In Process Citation]. *Arterioscler. Thromb. Vasc. Biol.,* **21,** 905–910.

[14] Yamamoto, Y., Yamagishi, S., Yonekura, H., Doi, T., Tsuji, H., Kato, I., Takasawa, S., Okamoto, H., Abedin, J., Tanaka, N., Sakurai, S., Migita, H., Unoki, H., Wang, H., Zenda, T., Wu, P., Segawa, Y., Higashide, T., Kawasaki, K., and Yamamoto, H. (2000) Roles of the AGE-RAGE system in vascular injury in diabetes. *Ann. N. Y. Acad. Sci.,* **902,** 163–170.

[15] Bierhaus, A., Illmer, T., Kasper, M., Luther, T., Quehenberger, P., Tritschler, H., Wahl, P., Ziegler, R., Muller, M., and Nawroth,

P. (1997) Advanced glycation end product (AGE)-mediated induction of tissue factor in cultured endothelial cells is dependent on RAGE. *Circulation,* **96,** 2262–2271.

[16] Yeh, C., Sturgis, L., Haidacher, J., Zhang, X., Sherwood, S., Bjercke, R., Juhasz, O., Crow, M., Tilton, R., and Denner, L. (2001) Requirement for p38 and p44/p42 mitogen-activated protein kinases in RAGE-mediated nuclear factor-kappaB transcriptional activation and cytokine secretion. *Diabetes,* **50,** 1495–1504.

[17] Tanaka, N., Yonekura, H., Yamagishi, S., Fujimori, H., Yamamoto, Y., and Yamamoto, H. (2000) The receptor for advanced glycation end products is induced by the glycation products themselves and tumor necrosis factor-alpha through nuclear factor-kappa B, and by 17beta-estradiol through Sp-1 in human vascular endothelial cells. *J. Biol. Chem.,* **275,** 25781–25790.

[18] Saito, M., Morishita, E., Asakura, H., Jokaji, H., Uotani, C., Kumabashiri, I., Yamazaki, M., Aoshima, K., and Matsuda, T. (1996) [Analysis of behaviors of plasma tissue factor and tissue factor pathway inhibitor in patients with various diseases]. *Rinsho. Ketsueki.,* **37,** 794–798.

[19] Kario, K., Matsuo, T., Kobayashi, H., Matsuo, M., Sakata, T., and Miyata, T. (1995) Activation of tissue factor-induced coagulation and endothelial cell dysfunction in non-insulin-dependent diabetic patients with microalbuminuria. *Arterioscler. Thromb. Vasc. Biol.,* **15,** 1114–1120.

[20] Jinnouchi, Y., Sano, H., Nagai, R., Hakamata, H., Kodama, T., Suzuki, H., Yoshida, M., Ueda, S., and Horiuchi, S. (1998) Glycolaldehyde-modified low density lipoprotein leads macrophages to foam cells via the macrophage scavenger receptor. *J. Biochem (Tokyo).,* **123,** 1208–1217.

[21] Vlassara, H., Fuh, H., Donnelly, T., and Cybulsky, M. (1995) Advanced glycation end products promote adhesion molecule (VCAM-1, ICAM-1) expression and atheroma formation in normal rabbits. *Mol. Med.,* **1,** 447–456.

[22] Wang, H., Li, F., Runge, M., and Chaikof, E. (1997) Endothelial cells exhibit differential chemokinetic and mitogenic responsiveness to alpha-thrombin. *J. Surg. Res.,* **68,** 139–144.

[23] Jellinger, P., Dickey, R., Ganda, O., Mehta, A., Rodbard, H., Seibel, J., Shepherd, M., and Smith, D. (2000) The American Association of Clinical Endocrinologists: Medical guidelines for Clinical Practice for the diagnosis and treatment of dyslipidemia and prevention of atherogenesis. *Endocr. Pract.,* **6,** 162–213.

[24] Kornberg, A., Rahimi-Levine, N., Yona, R., Mor, A., and Rachmilewitz, E. (1997) Enhanced generation of monocyte tissue factor and increased plasma prothrombin fragment 1 + 2 levels in patients with polycythemia vera: Mechanism of activation of blood coagulation. *Am. J. Hemtol.,* **56,** 5–11.

[25] Iochman, S., Reverdiau-Moliac, P., Beaujean, S., Rideau, E., Lebranchu, Y., Bardos, P., and Gruel, Y. (1999) Fast detection of tissue factor and tissue factor pathway inhibitor messenger RNA in endothelial cells and monocytes by sensitive transcription–polymerase chain reaction. *Thromb. Res.,* **94,** 165–173.

[26] Ichikawa, K., Yoshinari, M., Iwase, M., Wakisaka, M., Doi, Y., Iino, K., Yamamoto, M., and Fujishima, M. (1998) Advanced glycosylation end products induced tissue factor expression in human monocyte-like U937 cells and increased tissue factor expression in monocytes from diabetic patients. *Atherosclerosis,* **136,** 281–287.

[27] Reverter, J., Reverter, J., Tassies, D., Rius, F., Monteagudo, J., Rubies-Prat, J., Escolar, G., Ordinas, A., and Sanmarti, A. (1997) Thrombomodulin and induced tissue factor expression on monocytes as markers of diabetic microangiopathy: A prospective study on hemostasis and lipoproteins in insulin-dependent diabetes mellitus. *Am. J. Hematol.,* **56,** 93–99.

[28] Zumbach, M., Hofmann, M., Borcea, V., Luther, T., Kotzsch, M., Muller, M., Hergesell, O., Andrassy, K., Ritz, E., Ziegler, R., Wahl, P., and Nawroth, P. (1997) Tissue factor antigen is elevated in patients with microvascular complications of diabetes mellitus. *Exp. Clin. Endocrinol. Diabetes,* **105,** 206–212.

[29] Carr, M. (2001) Diabetes mellitus: A hypercoagulable state. *J. Diabetes Complications.,* **15,** 44–54.

[30] Stitt, A., He, C., and Vlassara, H. (1999) Characterization of the advanced glycation end-product receptor complex in human vascular endothelial cells. *Biochem. Biophys. Res. Commun.,* **256,** 549–556.

[31] Saishoji, T., Higashi, T., Ikeda, K., Sano, H., Jinnouchi, Y., Ogawa, M., and Horiuchi, S. (1995) Advanced glycation end products stimulate plasminogen activator activity via GM-CSF in RAW 264.7 cells. *Biochem. Biophys. Res. Commun.,* **217,** 278–285.

[32] He, C., Koschinsky, T., Buenting, C., and Vlassara, H. (2001) Presence of diabetic complications in type 1 diabetic patients correlates with low expression of mononuclear cell AGE-receptor-1 and elevated serum AGE. *Mol. Med.,* **7,** 159–168.

[33] Crossman, D., Carr, D., Tuddenham, E., Pearson, J., and McVey, J. (1990) The regulation of tissue factor mRNA in human endothelial cells in response to endotoxin or phorbolester. *J. Biol. Chem.,* **265,** 9782–9787.

[34] Tuttle, K., Puhlman, M., Cooney, S., and Short, R. (1999) Urinary albumine and insulin as predictors of coronary artery disease: An angiographic study. *Am. J. Kidney. Dis.,* **34,** 918–925.

[35] Lunetta, M., Barbagallo, A., Attardo, T., Crimi, S., and Sangiorgio, L. (1996) Frequency of coronary heart disease and related risk factors in a diabetic and nondiabetic population: A comparative study. *Panminerva. Med.,* **38,** 211–216.

[36] Napoleone, E., Di Santo, A., Camera, M., Tremoli, E., and Lorenzet, R. (2000) Angiotensin-converting enzyme inhibitors downregulate tissue factor synthesis in monovytes. *Circ. Res.,* **86,** 139–143.

